# Dental Fallacies

**Published:** 1880-01

**Authors:** John J. R. Patrick


### AMERICAN JOURNAL
OF
### DENTAL SCIENCE.

Vol. XIII. THIRD SERIES—JANUARY, 1880. No. 9.

### ARTICLE I.

### *Dental Fallacies.*

BY JOHN J. R. PATRICK, D. D. S.

[An address delivered before the Missouri State Dental Association, held at Sweet Springs, June, 1879.]

The paper which I have the honor to present to you this evening, will embrace but a small part of a large but unexplored field, and will simply aim to point the way to others who may feel disposed to investigate the truth or fallacy of the "Degeneracy of the Human Teeth." I know, gentlemen, that you are well aware of the deficiency of information possessed by intelligent people, as well as the lack of knowledge by our medical brethren in regard to the errors connected with our profession.

In the discussion of this subject it will be necessary to examine some of the fallacies which the literature of our profession discloses, because it is from this class of literature alone that we must look for the most disastrous results when in error, and for the greatest good when strictly scientific. At the outset it will be necessary to ascertain, if possible, the origin of the general belief in the degeneracy of man, including his teeth. Long before science arose to deal with this subject, this belief in degeneracy became enwoven with our history, our habits and our creeds; all the errors in regard to diseases with which man appears to be afflicted, rests on the Mosaical account of the fall, which was only meant to account for the apparent disorders existing in nature. A teleological idea which is essentially bound up with the other idea—that man was created in order that the world might not be without some intelligent creature who could appreciate it. But the countless ages that elapsed prior to the appearance of man is a sufficient answer to the philosophical theories of final causes.

We can trace the spirit of this doctrine of degeneracy through successive ages down to our own time. It has been maintained and defended from every pulpit and rostrum in Christendom, and forms the basis of all opposition to every advance in every department of natural science, especially those branches which relate to Anthropology or the science of man. Like predestination in Ethics, it has a constant tendency to paralyze every intellectual effort. These opinions are simply dogmatic and traditional, held by this man because it has been held by that man, a generality of assent, which means but little more than a multiplicity of credulities. There is nothing so servile as the human mind in the presence of established or received opinions, and this condition is not confined to the uneducated in the delicate art of observation, for men of eminent culture find great difficulty sometimes in seeing plain facts when they come in conflict with their preconceived notions. But the questioning spirit of modern science demands more than a mere belief. It requires a careful classification of existing facts and their relations to each other. It scorns to float on the stagnant pools of common opinion; it demands facts, numerous and plain, and the conclusions from them inevitable. These must precede all theories, all attempts at exposition; the verdict must come after the evidence, and not before it. In the interest of science, therefore, it seems to me that some protest is now called for against the doctrine promulgated at this late date from so high and honorable a position as a professor's chair in one of our dental colleges. I allude to the opinions expressed in an essay written by Prof. Henry S. Chase, of St. Louis, on causes of the degeneracy of the teeth and published in a valuable little work issued in numbers by the publishing house of Estis & Lauriat, of Boston.

In this essay, the Professor asserts that foreigners who have been here only two or three years often go to the dentist and say, "My teeth are getting bad; they never decayed until I came to America. There must be something in the climate which injures them." He further says: "I believe that climate has nothing to do with decay. The savages who inhabited the State of Missouri one hundred years ago, or even fifty years since, had teeth perfectly formed and of dense structure and undecayed."

The Professor unfortunately fails to furnish any better reason than the foreigner, or any kind of proof but his simple assertion. He certainly did not examine the teeth of these savages, and he therefore was in duty bound to give some good reason for the statement. He states further: "I have in my possession a large number of teeth picked up out of the earth by *myself* five years ago from the bottom of a mound on the banks of the Mississippi which was being removed. None of these teeth bear marks of decay! The red man of the last three hundred years has always had the reputation of perfect dental structures." The reasons the Professor furnishes are as follows: The food and habits of these savages were simple and natural. A large number of isolated teeth (it would be desirable to know how many) picked up from the bottom of a demolished mound, and the bare reputation that the savage enjoyed for sound teeth, during the last three centuries, justifies the Professor in the conclusion that it was his natural mode of living that secured him the blessings of a sound set of teeth.

Hence, unnaturally prepared food and not climate is the cause of dental decay. The Professor again states that "the common field hands of the South usually had strong, well-formed and undecayed teeth; while the house servants were more or less afflicted with the toothache, like their masters and mistresses. The food of the field hands was simple; while that of the house servants was the reverse and more like that of the whites."

The Professor, in this statement concerning the negro's teeth, I am well aware is on the strong popular side of the question. The great contrast between the negro's skin and his teeth is a powerful factor in this legend; for a negro's teeth must be very dirty and bad indeed not to appear white to the casual observer. The color and prognathism of the true negroes, and the consequent obliquity of implantation of the incisors, together with a receding chin, gives to his teeth great prominence. In this particular he differs but little from all the other low types of mankind. But the field hands of the South and the house servants were two very different people—the house servants, as a rule, had more or less European blood in their veins, which together with association, made them more intelligent and better fitted for places of trust than their purer-blooded brethren of the field. Only so far as mixed races are more subject to irregularities of the teeth, did the house servants suffer more from diseased teeth than the field hands. Before our late civil war, the teeth of the house servants in the South were attended to as strictly and with as little regard to expense as the teeth of the rest of the family. Aside from other considerations, an aching tooth in the household, whether in the mouth of master, mistress or slave, was a very unpleasant association, and a recurrence was guarded against by a visit to the family dentist. The field hands, on the contrary, when afflicted, disturbed no one excepting a few sympathizing friends in his quarters, who furnished the patient with all the nostrums and charms of which they were capable, for the sufferer, in most cases, dreaded the turnkeys of the plantation physician with a child-like horror. A visit to a dental office by a field hand was out of the question. A residence in the South before the war and a good share of Ethiopian practice (all extracting) justifies me in saying that the negro's teeth, beyond the age of twenty, are uniformly bad, and the mulatto's worse. If an examination were made to-day of all the negroes' mouths over forty years of age, by far the greater majority would be found approaching or in the condition of the venerable "Uncle Ned's," which has been recorded in that simple but pathetic ballad which bears his name. The Professor further states that people in the rural districts, one hundred years ago, had far better teeth than their descendants, and that when he was a boy, toothache was not common. Many persons eighty years of age had never lost a tooth. (Query: Did the boy examine the aged people's mouths in order to make this statement?) And he further states that Irish girls who come to this country for service usually have good teeth, but in two or three years their teeth decay surprisingly. This is easily accounted for (says the Professor) when it is notorious that they eat large quantities of food made from superfine flour, of which they rarely tasted in their native country. Armed with these unsubstantial theories, the Professor looks to other influences than climate for the degeneracy of the teeth, and finds the cause of all the mischief in the use of XXX superfine wheaten flour; so that the teeth of fine-flour eaters are defective, their children inherit their defective dental organization, and so the mischief spreads!

Happily for the *bone* if not the sinew of the country, unbolted flour is not the only source from which man obtains his supply of lime salts for his bone tissue. A very little reflection will show that there is more lime salts in solution in one quart of spring water, than can be obtained from the bran and short in one bushel of wheat. The water we drink and other food containing lime salts, will more than compensate the loss of bran and shorts taken from the wheat by the miller. For my own part, I apprehend but little danger of the prospective American appearing eventually boneless and devoid of teeth. At least it must be shown that the average American lacks in bone tissue before this hypothesis can be entertained. It must be shown that the average Bavarian, who lives on rye bread and unbolted flour, has larger bones than the proverbially "raw-boned Yankee." There must first be proof that a calf fed on bran and shorts will have larger bones and teeth when grown than one turned out to grass. So long as the secretions of the body deposit calculi and carry from the system large quantities of lime salts, there need be no fears of succeeding generations becoming edentulous.

I am well aware that it is, and has been the fashion of the times to charge deterioration of man to the advance of centuries. It is safe to do so; it is also orthodox. "Man is growing weaker and wiser," says one. "People do not live as old now as formerly," says another. "My great-grandfather never wore spectacles," says a third. "My great-grandmother never lost a tooth, and she was over eighty," says a fourth. And yet if you were to ask either of the two last named the Christian names of either grandfather or grandmother, they would not be able to tell you. Yet they will profess to know the condition of their teeth and eyes. If this degeneracy of the human family were true, it ought also to be true of domestic animals, for the same laws which govern the physical condition of the one likewise govern the condition of the other. All animals placed in conditions favorable to their development increase in size, strength and beauty, and it cannot be otherwise with man.

Men of a purely classical education have contributed much to strengthen the belief in the degeneracy of man; they have a mania for measuring every species of excellence by a Greek and Roman standard, and are attached to the past by a ligature which they are incapable of severing. They are lost in admiration at the sagacity and almost intuitive wisdom evinced in the writings of such men as Aristotle, Plato and Hippocrates, and having but a slight acquaintance with the progress made in natural science of modern times themselves, deem the superficial knowledge of the ancients equal if not superior to the moderns. Such a man is the Boston Demosthenes (Wendell Phillips)—"a man of swift and tuneful tongue," who has frequently treated the public, in form of lecture, to a plaintive lamentation over the lost arts. He has stated in this oft-repeated lecture that the teeth of Egyptian mummies have been found to contain gold fillings. This very important statement, if founded in fact, in the place of being drawn from the pen of some sensational newspaper reporter, would only prove that teeth decayed then as now; but I am far from giving the least credit to such a statement. Such marvelous theories might become a lawyer or an orator to sustain a false but popular lecture, but would poorly serve the interests of science, which has little to do with the dreamland of conjecture and speculation. I have watched the current literature on this question for several years, and the result has been to convince me that the prejudice which I am combatting is so deeply rooted in most minds, that it would be very difficult to meet the assertions made by members of our own profession, were it not for the positive evidence which I shall present to you this evening. The general literature of the past, as might be expected, furnishes as little on the subject of decayed teeth and toothache as the current literature of the present day. The Bible, for instance, speaks of teeth broken, teeth put on edge, and in the Jewish law of retaliation, the loss of one tooth is made to satisfy the loss of another. The nearest approach to a knowledge of bad teeth is shown in one of Solomon's love songs, where he compares the teeth of the subject of his inspiration to a flock of sheep that are even shorn and fresh from the washing; which simply shows that such teeth were unusual, or he would not have selected them as special objects of admiration. The Roman satirical poet, Juvenal, who flourished about the seventieth year of our era, in his fifth satire, describing the common custom at Roman feasts of serving the guests according to their rank with fine and indifferent food at the same table, says:

> "The scoundrel hands the bread you scarce can break—
> Hard, musty lumps, which make the grinders ache."

To enumerate the many silly charms for the cure of the tooth-ache in the early history of medicine would extend this essay to an indefinite length. In the middle ages the saints were fearful enemies to the progress of the science of medicine. There was not a limb of the human body but had its special guardian saint; no disease, pain or accident but an especial saint was appealed to for relief, and aching teeth were well provided for. Saint Apollonia, Saint Petronilla and Saint Lusy were the celestial dentists.

Paracelsus, the great charlatan of Germany, who rose to such popular fame in the early part of the sixteenth century that he obtained the professorship of medicine at Basil, and whose remedies consisted principally of magic, astrology and geomancy, gave cabalistical words to be repeated for the cure of the tooth-ache. In the early part of the seventeenth century Bishop Hall, in one of his philippies against the ignorance of some of his enemies, says "that charms are their physicians, and they wear Paracelsian characters for the tooth-ache." In a satirical poem written by the same author occurs the following verse:

> "Or Gallia wore a velvet mastic patch
> Upon her temples when no tooth did ache."

A nail driven into an oak tree is a very ancient remedy for the tooth-ache, and is resorted to in the rural districts of Europe to-day. I had the good fortune to see the operation performed successfully by a Swiss before an admiring crowd of farm hands, and he declared to me, through his interpreter, that the remedy had never failed him. But by far the finest record of the loss of teeth and the existence of tooth-ache near three centuries ago, is to be found in the works of that most illustrious of dramatic poets, William Shakespeare, for had tooth-ache and the subsequent loss of teeth not been a natural condition and a common malady, he, above all men, would not have mentioned it. I find in the comedy of "As You Like It," in the scene where the brothers Oliver and Orlando quarrel, the old servant, Adam, interferes, but is rebuked and called an old dog by the elder brother. To which Adam replies: "Is old dog my reward? Most true, I have lost my teeth in your service; God be with my old master, he would not have spoken such a word." In the same play Adam says he is nigh eighty years old. In the same comedy, act II, scene 7th, Jacques compares the world to a "stage—the men and women merely players; man in his time plays many parts," his acts being seven ages. The 7th and last scene, that ends this strange, eventful history, is second childishness and mere oblivion: *sans* teeth, *sans* eyes, *sans* taste, *sans* everything. In the 1st scene of the 5th act of the comedy of "Much Ado About Nothing," two old men, Leonato and Antonio (brothers) are introduced, Leonato grieving over the death of his daughter, who has been slandered to death by one Claudio. His brother, Antonio, proffers him consolation, to which Leonato replies: "I pray thee peace; I will be flesh and blood; for there was never philosopher that could endure the tooth-ache patiently." Further on in the same scene the two old men confront the traducer and challenge him to mortal combat, when it is stopped by the Prince of Arragon, and the two old men leave breathing vengeance. At their exit Benedick enters, and is informed by the Prince that he almost came in time to part a fray, and Claudio says: "We had like to have had our two noses snapped off by two old men without teeth." In the same play, act III, scene 2nd, Benedick (who is supposed by his companions to be in love) appears with the tooth-ache, when the following conversation takes place:

*Benedick.*—I have the tooth-ache.

*Don Pedro.*—Draw it.

*Benedick.*—Hang it.

*Claudio.*—You must hang it first and draw it afterwards.

*Don Pedro.*—What! Sigh for the tooth-ache?

*Leonato.*—Where there is but a humor or a worm?

*Benedick.*—Well, every one can master a grief but he that hath it.

*Claudio.*—Yet say I, he is in love.

*Benedick.*—Yet is this no charm for the tooth-ache.

In the tragedy of Cymbeline, the gaoler says to Posthumus: "Indeed, sir, he that sleeps feels not the tooth-ache."

In the comedy of the "Two Gentlemen of Verona," Launce produces a catalogue of the virtues and vices of his mistress, which Speed, another clownish servant, reads for him. Among the vices enumerated are the following:

*Speed.*—Item: she hath no teeth.

*Launce.*—I care not for that neither, because I love crusts.

*Speed.*—Item: she is curst.

*Launce.*—Well, the best is, she hath no teeth to bite.

In the latter part of the seventeenth century Dean Swift, in a satirical poem entitled "A Beautiful Nymph Going to Bed," gives a very minute description of all the artificial adornments in use in his day, and which his Nymph removes before retiring. In the catalogue enumerated is artificial teeth.

> "Now dext'rously her plumpers draws,
> That serve to fill her hollow jaws;
> Untwists a wire, and from her gums
> A set of teeth completely comes."

The poet Robert Burns bears testimony to the existence of decayed and aching teeth in Scotland in his immortal little poem an "Address to the Tooth-ache," written soon after the author had been tormented with that disorder. One stanza will be sufficient to show the author's appreciation of it:

> "When fevers burn or ague freezes,
> Rheumatics gnaw, or colic squeezes,
> Our neighbor's sympathy may ease us,
> Wi' pitying moan;
> But thee, thou hell o' a' diseases,
> Aye makes our groan."

Any person but slightly acquainted with European art must know that Dutch, German and Flemish painters have all given ludicrous scenes of tooth extraction, which is a sufficient answer in itself to that pleasing legend which foreigners are so fond of telling "That they never heard of bad or aching teeth in the old country." If this will not suffice, ask the barber-surgeons, the German ones; they who were licensed to practice their calling in Germany, and they will tell you that tooth extraction was included in the list of the operations they were qualified to perform.

The expression of firmness can not be depicted on the human countenance without the teeth, while their loss is the very type of feebleness. Painters, poets and sculptors at all times, in depicting or describing old age, have always portrayed their subjects without teeth. The sunken lips, protruding chin and drooping nose—no hard or fixed expression of firmness can be produced when they are gone; the face becomes flaccid and yielding, and this in proportion to the loss sustained in alveola, for if the teeth are lost early in life, the absorption becomes complete, and the result is the loss of the attachment of the principal muscles of expression. I do not feel it necessary to dwell further on the evidence capable of being brought forth from the literature of the historic period in support of the truths already presented; opportunity will be constantly afforded members of our profession, in the course of their general reading, to add largely to the evidence I have presented. Poor as the evidence may be that I have furnished from the writings of men, the evidence furnished by the remains of the prehistoric man is unmistakable, silent but eloquent in its silence, and beyond all controversy.

In the Academy of National Sciences of Philadelphia, there is a large collection of human crania taken from every portion of the known world. I refer to that magnificent collection made by Dr. Samuel George Morton. These shulls form the basis of his large work entitled "Crania Americana, or a Comparative View of the Skulls of Various

Aboriginal Nations of North and South America." I have spent some time with these human remains, and while they are very deficient in teeth, many being lost, there are enough remaining to show that tooth decay and the maladies consequent thereto was not uncommon with these different races of men. In a work on Human Anatomy, by Alexander Monroe, published at Edinburgh, in 1813, I find on page 352, vol. I, a table of measurement of British skulls where there were no teeth, and the alveolar completely absorbed. I have in my own collection of American crania taken from the mounds of the American Bottom in St. Clair and Madison Counties, Illinois, nearly one hundred well prepared skulls of the so-called mound builders, and it is the exception to find among the whole number a sound set of teeth. In obtaining this number of comparatively good crania not less than four hundred graves were opened, the rest being too frail to preserve. The marks of alveolar abscess are common; loss of molars and bicuspids are frequent, with complete absorption of the sockets. I have two cases of antral abscess, with loss of the external wall of the antrum in one of them. I have one case of entire loss of teeth, with absorption so complete that the mental foramina is obliterated and the nasal process entirely gone. I have a number of cases in which the crowns of the teeth are in all stages of decay; others with the fangs or roots remaining in the sockets, the crowns gone entirely. I have but two skulls in which the front teeth lap over each other; in all the other cases the masticating surface of the upper jaw fits perfectly that of the lower, and so with all the teeth that are not missing. The incisive te th do not lap, but impinge on each other at their cutting edges like the molars, and are worn quite flat, so that when we look along the surface of mastication we perceive that it is almost a perfect plane. This appears to be the case with all primitive races, for they eat their food in quite a different manner from what we do. There are, however, exceptional persons in modern times who use their teeth in the ancient way. The teeth of the primitive man were used more for prehension than with us. Cuvier says, in his Comparative Anatomy, that the ancient Egyptians used their incisive teeth for the same purpose, for the incisive teeth of the mummies are all truncated, and with flat coronals. The teeth of the ancient British and Gaulish skulls are in the same condition. The introduction of the knife and fork or chopsticks in modern times, to convey food to the mouth, has no doubt modified the form of the jaws and the antagonism of the teeth.

Whatever may be said in regard to the difference of antagonism in the jaws of the ancients, the savages and the civilized man of modern times, there can be no difference of opinion as to the certainty of disease and death, for the evidence is beyond dispute that if there is a law in nature more constant than any other, it is decay and death, either in part or in mass. Every germ, in its development, meets with obstacles, meets with contending forces; so that countless millions of germs or seeds that are produced, rot before ripening and never come to maturity, and by far the largest number that do mature are warped, gnarled and twisted to such an extent that but few can be said to be in that condition which physiologists call normal. Constant, unrelenting and persistent are the contending forces which govern life; an excess of either one or the other must change and modify the character of the organism upon which they act; and the wonder is to the observant mind, not that nature does her work so poorly, but that she does it so well.

In conclusion, gentlemen, I desire to say that my only object in criticising the writings of others is prompted by an earnest desire to see the science of dentistry placed in its true position, alike beyond the reach of ridicule, fraudulent imitation and scientific dandyism.*

---

[*At the close of the essay a motion was made to examine the teeth of all the negroes in the employ of the Sweet Springs Company, and a committee was appointed for that purpose. The committee, after a careful examination of over thirty negroes, reported their teeth in a uniformly bad condition.]

